# Supplementary material for: Cardiovascular Outcomes of ST-Elevation Myocardial Infarction (STEMI) Patients without Standard Modifiable Risk Factors (SMuRF-Less): The Intermountain Healthcare Experience
Source: J Clin Med. 2022 Dec 22;12(1):75. doi: 10.3390/jcm12010075 (PMC9821748; doi:10.3390/jcm12010075)
Supplement: Supplementary file 1 [file jcm-12-00075-s001.zip › jcm-2080912-supplementary.pdf]

Supplement Contents

Table S1: Baseline Characteristics of STEMI Patients by SMuRF Count

Table S2: Interventions and Medications of STEMI Patients by SMuRF Count

Table S3: Outcomes of STEMI Patients by SMuRF Status with Former Smoking Not Included as a Risk Factor

Table S1: Baseline Characteristics of STEMI Patients by SMuRF Count

Demographics and Clinical Characteristics	SMuRF Count 0 n=919		1 n=834		2 n=1007		3 n=649		4 n=101		P-value
	n	%	n	%	n	%	n	%	n	%	
**Age, median (IQR)**	61 (52, 70)		60 (52, 69)		62 (54, 72)		62 (54, 71)		59 (52, 67)		0.09
**Age groups**											0.005
<40	49	5.3%	31	3.7%	31	3.1%	18	2.8%	5	5.0%	
40-49	140	15.2%	138	16.6%	128	12.7%	80	12.3%	14	13.9%	
50-59	228	24.8%	245	29.4%	276	27.4%	166	25.6%	33	32.7%	
60-69	271	29.5%	216	25.9%	269	26.7%	200	30.8%	32	31.7%	
70-79	150	16.3%	130	15.6%	199	19.8%	131	20.2%	11	10.9%	
>79	80	8.7%	74	8.9%	104	10.3%	54	8.3%	6	5.9%	
**Sex**											0.03
Male	709	77.2%	615	73.7%	744	73.9%	455	70.1%	71	70.3%	
Female	210	22.9%	219	26.5%	263	26.1%	194	29.9%	30	29.7%	
**Race**											0.03
White/Caucasian	818	89.0%	734	88.0%	899	89.3%	544	83.8%	83	82.2%	
African American	8	0.9%	5	0.6%	5	0.5%	4	0.6%	0	0.0%	
Asian	15	1.6%	19	2.3%	20	2.0%	18	2.8%	0	0.0%	
Pacific Islander	3	0.3%	2	0.2%	2	0.20%	1	0.2%	0	0.0%	
Unknown	75	8.2%	74	8.9%	81	8.0%	82	12.6%	18	17.8%	

**Family history of heart disease**	137	14.9%	237	28.4%	334	33.2%	221	34.1%	57	56.4%	<0.0001
**Comorbidities**											
Atrial Fibrillation (AF)	112	12.2%	119	14.3%	167	16.6%	121	18.6%	13	12.9%	0.005
COPD	52	5.7%	54	6.5%	91	9.0%	74	11.4%	15	14.9%	<0.001
Depression	136	14.8%	129	15.5%	153	15.2%	141	21.7%	15	14.9%	0.002
Heart Failure (HF)	33	3.6%	26	3.1%	48	4.8%	47	7.2%	5	5.0%	0.002
Stroke	12	1.3%	7	0.8%	16	1.6%	13	2.0%	1	1.0%	0.40
**SMURF Criteria**											
Diabetes	0	0%	291	34.9%	172	17.1%	480	74.0%	101	100%	NA
Hyperlipidemia	0	0%	136	16.3%	755	75.0%	621	95.7%	101	100%	NA
Hypertension	0	0%	182	21.8%	832	82.6%	621	95.7%	101	100%	NA
Smoking history											NA
Never	919	100%	609	73.0%	752	74.7%	424	65.3%	0	0%	
Former	0	0%	69	8.3%	93	9.2%	76	11.7%	47	46.5%	
Current	0	0%	156	18.7%	162	16.1%	149	3.0%	54	53.5%	

Table S2: Interventions and Medications of STEMI Patients by SMuRF Count

Treatments and Medications	SMuRF Count 0 n=919		1 n=834		2 n=1007		3 n=649		4 n=101		P-value
	n	%	n	%	n	%	n	%	n	%	
PCI performed	778	84.7%	721	86.5%	897	89.1%	562	86.6%	87	86.1%	0.24
CABG	58	6.3%	55	6.6%	78	7.8%	65	10.0%	12	11.9%	0.02
**Discharge Medications**											
Beta Blocker	787	85.6%	699	83.8%	849	84.3%	555	85.5%	79	78.2%	0.31
ACE-I / ARB	576	62.7%	544	65.2%	678	67.3%	454	70.0%	63	62.4%	0.03
Anticoagulant	333	36.2%	280	33.6%	339	33.7%	231	35.6%	37	36.6%	0.68
Antiplatelet	887	95.7%	797	95.6%	968	96.1%	628	96.8%	97	96.0%	0.78
Aspirin	875	95.1%	787	94.4%	958	95.1%	620	95.5%	96	95.0%	0.88
Calcium channel blocker	100	10.9%	63	7.6%	126	12.5%	117	18.0%	8	7.9%	<0.0001

Table S3: Outcomes of STEMI Patients by SMuRF Status with Former Smoking Not Included as a Risk Factor

	SMuRF n=2522		No SMuRF n=988					
	**n**	**%**	**n**	**%**	**Unadjusted p-values**	**Adj** HR**	**95% CI**	**p-value**
**60-day Outcomes**								
MACE	207	8.2%	74	7.5%	0.52	0.93	(0.71, 1.21)	0.58
Death	168	6.7%	67	6.8%	0.87	1.02	(0.77, 1.36)	0.88
MI	14	0.6%	4	0.4%	0.58	NA*		
HF Hospitalization	26	1.0%	3	0.3%	0.04	NA*		
**Long-term Outcomes**								
MACE	795	31.5%	215	21.8%	0.002	0.81	(0.69, 0.94)	0.005
Death	631	25.0%	175	17.7%	0.02	0.84	(0.71, 1.00)	0.05
MI	175	6.9%	45	4.6%	0.11	0.80	(0.57, 1.11)	0.18
HF Hospitalization	123	4.9%	15	1.5%	0.0001	0.37	(0.21, 0.63)	0.0003

Analysis: Cox proportional hazard regression was used to examine outcomes adjusted for baseline differences comparing No SMuRF vs SMuRF.
Adj**= No SMuRF vs SMuRF adjusted for age, sex, ACE-I / ARB, AF, COPD, family history
*NA = no modeling done due to too few outcomes.
ACE-I = angiotensin converting enzyme inhibitors; AF = atrial fibrillation; COPD = chronic obstructive pulmonary disease; HF = heart failure; MACE = major adverse cardiovascular event; MI = myocardial infarction; HR=hazard ratio
